# Research on patients with multiple health conditions: different constructs, different views, one voice

**DOI:** 10.15256/joc.2011.1.11

**Published:** 2011-12-27

**Authors:** Jose M. Valderas, Stewart W. Mercer, Martin Fortin

**Affiliations:** ^1^Health Services and Policy Research Group, Department of Primary Care Health Sciences, NIHR School for Primary Care Research, University of Oxford, Oxford, UK; ^2^General Practice and Primary Care, Institute of Health and Wellbeing, University of Glasgow, Glasgow, UK; ^3^Department of Family Medicine, Université de Sherbrooke, Sherbrooke, Québec, Canada

**Keywords:** Comorbidity, multimorbidity, multiple health conditions

Technological advances, improvements in medical care and public health policies have resulted in a growing proportion of patients with multiple health conditions. The prevalence of multiple health conditions among individuals increases with age, is substantial among older adults, and will increase dramatically in coming years [1–4]. This phenomenon has received growing interest in the most recent literature and has led to several – and often differing – conceptualizations.

The term ‘comorbidity’ was originally defined by Feinstein as “any distinct additional clinical entity that has existed or may occur during the clinical course of a patient who has the index disease under study” [5]. This definition places one disease in a central position and all other condition(s) as secondary, in that they may or may not affect the course and treatment of the index disease [6]. Feinstein’s principle has been applied all too readily as if the effect of comorbidity was secondary or indeed negligible. In clinical research, individuals with a narrowly defined index condition and no major comorbidities are usually enrolled, leaving the majority of the patients seen in a typical family practice [7, 8] out in the cold. In clinical practice, management of the index condition invariably takes priority, with disjointed – if any – treatment plans developed for each of the comorbidities [6]. This model of care is typical of delivery systems constructed around specialized care, where areas of expertise are defined around specific conditions and bodily systems [11]. Not surprisingly, clinical practice guidelines arising from that model of care lack pertinence for patients with multiple health conditions [9, 10].

The term ‘multimorbidity’ has emerged as a modern alternative to ‘comorbidity’. In this more ‘democratic’ approach, no particular condition is privileged over any other. Multimorbidity has been simply defined as the co-existence of two or more conditions. van den Akker [12] devoted substantial effort to providing the theoretical and empirical underpinnings of this concept, further expanded by Boyd and Fortin [6] and the International Research Community on Multimorbidity [13]. Consistent with the ‘generalist approach’ [14], the concept has been readily embraced by the research community in the areas of primary care, family medicine, and general practice. The concept of multimorbidity offers two main attractions: first, it implies that care delivery models should be centred around the patient as a whole, and not simply in relation to the presence of specific conditions; and second, it accommodates the differing trajectories of conditions – what the condition of interest is may be different for one individual at different moments in his/her life.

Both terms, ‘comorbidity’ and ‘multimorbidity’, focus on the presence of conditions, but it is not clear what a ‘condition’ actually may be [15]. Is hypertension a disease or a risk factor? In Western health systems, the differences between the management of diseases, on the one side, and prevention and risk factor management, on the other, are increasingly blurred. Thus, there is a need for researchers to operationally define their area of investigation each time new research is planned. Prevalence results are particularly prone to variation, depending on the list of diseases or conditions considered [16–18].

An additional limitation in regard to both of these constructs is that they do not take disease severity into account, hence the emergence of the morbidity-burden construct [11]. Intuitive as it seems, incorporating the notion of severity immediately raises the thorny issue of who should determine severity. Is it the clinician? Is it the patient? Or is it the health system? Measuring the severity of someone with chronic obstructive pulmonary disease using lung function measurements versus quality-of-life measurements or the cost of provision of care based on emergency department and inpatient admissions is very different. Finally, as many other factors may have to be considered in caring for patients with multiple health conditions, the need for a more holistic view has brought up the construct of patient complexity, taking into account socio-economic, cultural, environmental, and patient behaviour dimensions [11].

Research in the area of multiple health conditions is surprisingly scarce in comparison with research on specific diseases [7]. Research to date has largely focussed on epidemiology and analyses of the impact of multimorbidity on individuals and healthcare systems, with very few studies examining interventions to improve clinical outcomes [19]. There is little doubt, however, that the issue is moving up the international agenda [15, 20]. Promoting this area of research is timely as many healthcare systems are undergoing reforms and more attention is being given to disease management (particularly but not exclusively) in primary healthcare.

All the issues raised above are relevant to research on people with multiple health conditions. The journal has opted to use comorbidity in its name, and a number of well founded reasons explain this choice: for reasons of simplicity; in order to acknowledge both the relevance of research on comorbidity for the treatment of specific conditions and the historical pre-eminence of the construct; and finally, for an awareness of evolving concepts. We are looking forward to playing our part in promoting high-quality work in this research field and helping to develop comprehensive guidance on how best to manage individuals with multiple conditions using any of the current approaches. But this is only possible with your contributions, which we await with great interest.

As healthcare providers and researchers, we face important challenges in understanding and tackling the issues raised by multiple health conditions. However, the biggest challenges are faced on a daily basis by those we serve – the millions of people around the world living with multiple health conditions. We must work together, collaboratively, listening to each other’s views, reporting on their lived experiences, acting with them, and advocating for them. By joining with the people we call patients, as equal partners, we can build a common vision, inform each other, exchange ideas, and impart and receive knowledge and develop shared wisdom. The challenges ahead mean we must learn how to do things differently and better in the future, based on mutuality and respect. Together we can make a real difference by generating new evidence in this important research field and putting it into practice. Let us embrace the challenges, the different constructs, and the different views as one voice.
